# A kia ora, a wave and a smile: an urban marae-led response to COVID-19, a case study in manaakitanga

**DOI:** 10.1186/s12939-022-01667-8

**Published:** 2022-05-17

**Authors:** Cheryl Davies, Carmen Timu-Parata, Jeannine Stairmand, Bridget Robson, Amanda Kvalsvig, Dominique Lum, Virginia Signal

**Affiliations:** 1 Kōkiri Marae Hauora, Seaview, Wellington, New Zealand; 2 Te Rōpū Rangahau Hauora A Eru Pōmare, University of Otago, 23a Mein Street, Newtown, Wellington, New Zealand; 3 Department of Public Health, University of Otago, Wellington, New Zealand; 4 School of Medicine, University of Otago, Dunedin, New Zealand

**Keywords:** COVID-19, Health equity, Indigenous communities, Māori

## Abstract

**Background:**

When COVID-19 emerged, there were well-founded fears that Māori (indigenous peoples of Aotearoa (New Zealand)) would be disproportionately affected, both in terms of morbidity and mortality from COVID-19 itself and through the impact of lock-down measures. A key way in which Kōkiri (a Māori health provider) responded was through the establishment of a pātaka kai (foodbank) that also provided a gateway to assess need and deliver other support services to whānau (in this case, client). Māori values were integral to this approach, with manaakitanga (kindness or providing care for others) at the heart of Kōkiri’s actions. We sought to identify how Kōkiri operated under the mantle of manaakitanga, during Aotearoa’s 2020 nationwide COVID-19 lockdown and to assess the impact of their contributions on Māori whānau.

**Methods:**

We used qualitative methods underpinned by Māori research methodology. Twenty-six whānau interviews and two focus groups were held, one with eight kaimahi (workers) and the other with seven rangatahi (youth) kaimahi. Data was gathered between June and October 2020 (soon after the 2020 lockdown restrictions were lifted), thematically analysed and interpreted using a Māori worldview.

**Results:**

Three key themes were identified that aligned to the values framework that forms the practice model that Kōkiri kaimahi work within. Kaitiakitanga, whānau and manaakitanga are also long-standing Māori world values. We identified that kaitiakitanga (protecting) and manaakitanga (with kindness) - with whānau at the centre of all decisions and service delivery - worked as a protective mechanism to provide much needed support within the community Kōkiri serves.

**Conclusions:**

Māori health providers are well placed to respond effectively in a public-health crisis when resourced appropriately and trusted to deliver. We propose a number of recommendations based on the insights generated from the researchers, kaimahi, and whānau. These are that: Māori be included in pandemic planning and decision-making, Māori-led initiatives and organisations be valued and adequately resourced, and strong communities with strong networks be built during non-crisis times.

**Supplementary Information:**

The online version contains supplementary material available at 10.1186/s12939-022-01667-8.

## Background

*Me mahi tahi tatau, ka ora ai te iwi.*

*Mā ngā huruhuru e rere te manu.*

*We should work together, to enable the people to thrive.* *It is the feathers that support the bird to fly.*

Whakatauki (proverbs) [see Additional file 1 for Glossary of Māori language terms] represent the wisdom that guides Māori, the Indigenous people of Aotearoa (New Zealand). Whakatauki are most commonly used as inspirations in speeches but also as gentle reminders to steer everyday life [1]. The whakatauki above aligns with the values of Kōkiri Marae Hauora (Kōkiri), a large health and social service provider in the Hutt Valley region of Aotearoa [2] which is located on an urban marae (a traditional meeting place that is a vital part of everyday life for contemporary Māori). Kōkiri delivers a range of services utilising a community development model in conjunction with the medical model of healthcare [2]. At the heart of Kōkiri’s services are passionate kaimahi (workers) who utilise the core values of Kōkiri’s values framework [3] [see Additional file 2] to provide mana-enhancing (a way of engaging with others that enables them to retain their power and prestige) and unconditional manaakitanga (kindness, support, providing care for others) to any person who needs it.

Mana-enhancing ways of working were especially evident in Kōkiri’s response to their whānau (in this case, clients) during Aotearoa’s first national COVID-19 lockdown, which imposed strict nationwide stay-at-home orders from March 26 – May 13 2020 [4, 5]. Kia atawhai (Be kind) were words heard often in Aotearoa during this time. The Prime Minister Jacinda Ardern asked New Zealanders to be kind to each other in her daily media briefings:” Be strong but be kind. We will be ok” (17 March 2020), and the words were also often used in messaging around COVID-19, such as in street posters and electronic billboards. These words resonate with Māori and Māori health providers - such as Kōkiri - as it is how they operate, with manaakitanga woven through all that they do. Soon after Aotearoa’s first nationwide lockdown, we undertook a study to answer the following research question. What does an urban marae-led response to COVID-19 look like?

### The challenge that Kōkiri faced in early 2020

The COVID-19 elimination strategy successfully pursued by Aotearoa in 2020 meant there were relatively few COVID-19 cases in the community, [6] and one of the lowest mortality rates in the OECD [7]. However, when COVID-19 first emerged, there were fears that Māori would be disproportionately affected in terms of morbidity and mortality if community transmission of COVID-19 was allowed unchecked within Aotearoa [8–10]. These fears were well-founded. A lot of evidence pointed to Māori being more likely to experience significantly worse outcomes from COVID-19 than New Zealand Pākehā (New Zealand Europeans); Māori would likely have a higher risk of contracting COVID-19 in the first place, coupled with a higher risk of becoming unwell and of hospitalisation or death once contracted [8–11]. Conversely, there were fears that Māori would be disproportionately affected socially and economically through lock-down measures causing the loss of jobs and incomes [12] and that COVID-19 would increase the historically accumulated inequities and structural racism linked to colonisation and neoliberalism for Māori [13]. Again, these fears were well-founded and Kōkiri - as did other Māori health providers - quickly responded.

### Kokiri’s pandemic response

**Fig. 1:**
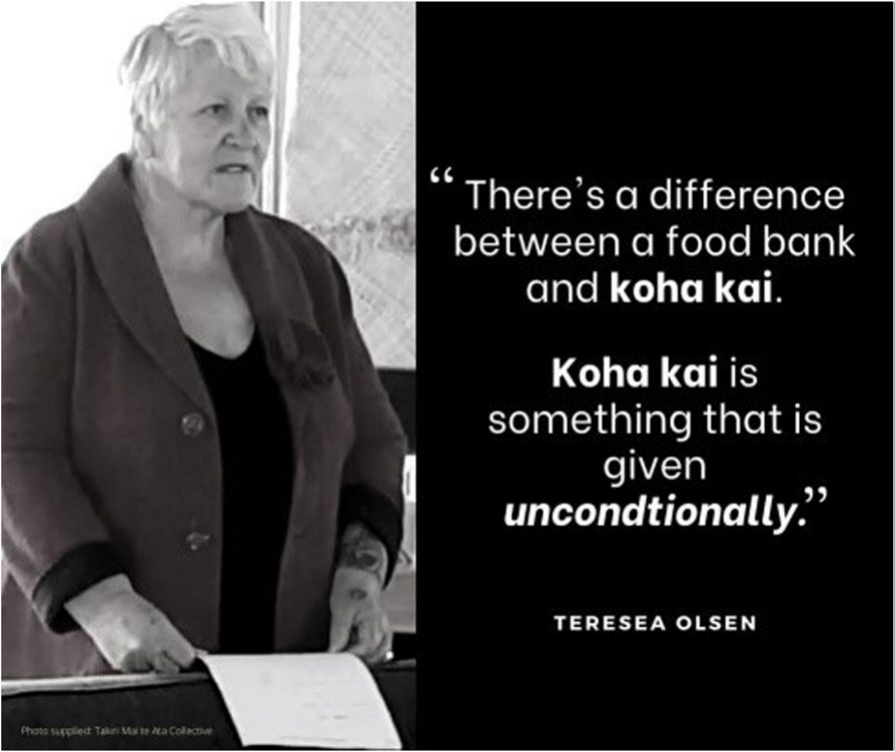
Koha kai

A key way in which Kōkiri responded was through the establishment of a pātaka kai (foodbank and community food resource) in Wainuiomata, a large - 20,000+ − but geographically isolated and predominantly low socioeconomic suburb in the Wellington region. Early in the pandemic, Kōkiri phoned all whānau on their books to assess their needs and realised there was an increasing unmet need for kai (food) within the Wainuiomata community. This was compounded as many mainstream foodbanks closed during the 2020 lockdown and those still operating often had strict access criteria or placed limits on the amount of food provided [14]. In contrast - and in keeping with the value of manaakitanga - Kōkiri’s leadership did not want barriers to accessing kai and so no criteria or limits were placed on the receipt of kai: if your whānau needed kai you received it. Neither did whānau wait in lines to access their kai parcel. Rather, all food parcels were delivered to the recipients’ door allowing for their mana to be upheld. Importantly, the pātaka kai was established as a community resource that provided back to the community (or koha kai). The Kōkiri General Manager - Teresea Olsen - describes the concept of koha kai as “something that is given unconditionally” (Fig. 1).

Kōkiri was also a crucial distribution mechanism for resources made available by government agencies and other organisations. Overall during the 6 weeks of the 2020 lockdown, 26,510 food parcels or cooked meals and over 60,000 frozen food packs were delivered to the doors of 31,115 whānau members. Households ranged in size from one to 22 people within one home (or in some cases motels which were used as emergency housing for homeless people). While the majority of whānau identified as Māori, large numbers were also Pākehā (17.8%) or Pacific peoples (14.7%). Importantly, the pātaka kai provided a gateway to allow kaimahi to assess whānau needs and to deliver many other services. These included providing blankets or warm clothing, medical support for people with long-term conditions, linking whānau to other health services or agencies, filling medication prescriptions and shopping for those unable to leave their house, giving influenza vaccinations, delivering sanitation packs and masks so that whānau could prevent the spread of Covid-19 and general support for whānau who were stressed or isolated.

### Māori-led disaster responses

Māori, Māori Health Providers and marae have often responded in public health crises. During the 2010 and 2011 Canterbury earthquakes, Te Rūnanga o Ngāi Tahu (an iwi - or tribe - in the South Island of Aotearoa) played a leading role in the response. This included mobilising health practitioners to deliver health care to those living in the hardest-hit communities, the establishment of a 24-h phone contact service, texting trees to ensure responders had up-to-date information and providing practical assistance such as food and shelter [15, 16]. Māori values were at the heart of this response. Kenney et al. [15] described this response as Māori using their collective knowledge to facilitate coping and resilience that strengthened the region’s disaster responsiveness and recovery. Similarly, following the 2016 Kaikōura earthquake Takakanga marae - located in Kaikōura - moved swiftly and effectively to become a registered Ministry of Civil Defence and Emergency Management Welfare Centre [17]. The marae provided a safe place to sleep for stranded tourists, served more than 10,000 meals and distributed 1700 care packages to their community [17]. In July 2016 amid a different public health crisis, Te Puea Marae - located in Auckland - opened their doors to people who were homeless, housing them on the marae and supporting them into permanent housing. By 2018, the Manaaki Tangata E Rua programme had supported 332 people to find or buy homes [18]. Again Māori values were integral, with manaakitanga at the heart of these marae-based interventions [18, 19].

### Motivation for this study

Despite the large number of Māori health and social service providers within Aotearoa evidence about how they operate and the impacts of their services is rarely documented. This paper provides a case study of how one urban marae-based Māori health provider operated under the mantle of manaakitanga (or Be Kind), during the 2020 national COVID-19 lockdown in Aotearoa.

## Methods

We used qualitative methods underpinned by kaupapa Māori methodology - both described below - to investigate the impact of contributions made by a Māori health provider to address the health and social support needs of Māori whānau in a pandemic. We used data from two sources: information from interviews held with Kōkiri whānau (clients) and focus groups held with Kōkiri kaimahi (workers). All interviews and focus group hui were undertaken between June and October 2020, soon after the 2020 lockdown restrictions were lifted.

### Kaupapa Māori methodology

This study was nested within kaupapa Māori research methodology in which Māori ways of knowing and doing are fundamental, Māori world views are normative and the goal is positive change to support improved Māori health and social outcomes [20–22].

Kaupapa Māori research is an indigenous Māori theoretical research framework that came about in reply to western research methodologies that privileged Western ways of knowing and thus undermined indigenous Māori knowledge and belief systems [22, 23]. This study was developed as a kaupapa Māori research study within a larger study investigating the COVID-19 pandemic and its response in Aotearoa [24]. It was carried out by indigenous Māori researchers utilising Māori beliefs and values and in which Māori identity, knowledge, and culture were central [22]. While kaupapa Māori research does not specify which research methods to utilise it does describe the guiding principles for research that align with Te Ao Māori (the Māori world view) and Māori realities [25]. Kaupapa Māori principles [22] guided all interactions with participants and the data generated by them. For example, whānau were invited to have a support person present, and in keeping with tikanga (protocol) karakia (prayer) to open and close the interview process was offered to all participants. The data was transcribed by Kōkiri administrative kaimahi - who understand Te Reo (Māori language) which was commonly used during the interviews and focus groups - and analysed by indigenous Māori researchers who also led the writing of this paper. In addition, Te Reo Māori has been privileged in the writing of this paper.

### Whānau (participant) interviews

The 26 participants interviewed about their lockdown experiences were whānau (clients) of Kōkiri. Two whānau were accompanied by a support person and their korero (feedback) was included in the interview transcripts and analysed. All participants were adults, ranging in age from early 20’s to early 80’s. Twenty-one were female (80.8%) and five male (19.2%), 23 (88.5%) self-identified as Māori, two as Samoan (7.7%) and one as New Zealand European (3.8%). Household size - or number of people in the household during the 2020 lockdown - ranged from one to 15 people, 10 (39%) increased in size, 12 (46%) remained the same, 3 (11.5%) decreased in size and one participant did not respond to this question. Sixteen participants were on a government benefit prior to lockdown and three lost their job during lockdown of whom two were receiving a government benefit at the time of interview. Fifteen whānau had chronic health conditions and five were caring for another whānau member in their household with a chronic condition.

### Whānau recruitment

Recruitment was carried out in Wainuiomata and Te Awakairangi (the Hutt Valley, a city in the Wellington Region of which Wainuiomata is a suburb). These communities were chosen because of the immense support provided by Kōkiri Marae to these communities during the 2020 nationwide COVID-19 lockdown. Whānau were identified and recruited through the Kōkiri pātaka kai database established to track support provided during the lockdown. A range of people was chosen to participate in this study with household size, type of support service received, age, and ethnicity considered when identifying potential participants. Three of the Māori researchers (CD, DL and HT) phoned participants and invited them to take part in the study. Once whānau agreed to participate, a time and place to interview were confirmed. All participants were given an information sheet about the project and signed a consent form prior to the interview commencing.

### Whānau interviews

The interviews took place within the context of whānaungatanga (relationships) established between Kōkiri Marae and the whānau that they serve. They were conducted soon after the lockdown was lifted, thus, while two whānau members were interviewed by telephone the remaining participants were interviewed kanohi-ki-te-kanohi (face to face) at venues they requested. These venues included their homes, the Wainuiomata Community Hub or Kōkiri Marae in the Hutt Valley. Data were collected through a semi-structured interview questionnaire (available from the authors on request). A Māori theoretical framework - Te Whare Tapa Whā [26] - was used to develop the questionnaire providing an enhanced exploration of the impacts of the COVID-19 lockdown on whānau spiritual, physical, mental and emotional wellbeing. Questions explored the impact of COVID-19 and the Government’s response on whānau, any issues and challenges whānau faced, how services responded to their needs, what helped them during the lockdown and how their 2020 lockdown experience could have been made easier.

The interviews ranged between 20 and 70 min in length, researcher field notes were taken and the interviews were audio-recorded and transcribed verbatim. Whānau received a grocery voucher and a COVID-19 sanitation pack (hand sanitiser, masks, gloves, and cleaning products) as an acknowledgement for their time. Ethical approval was obtained from the University of Otago Human Ethics Committee (Ref No. 20/079).

### Kaimahi focus groups

Two focus groups were held, one with eight kaimahi and the other with seven rangatahi (youth) kaimahi. Participants ranged in age from 14 to 55 years and were predominantly female. All self-identified as Māori.

### Kaimahi recruitment

A purposeful sample of Kōkiri kaimahi who set up and/or supported the pātaka kai operations - by packing and delivering kai parcels - during the lockdown was identified by the lead Māori researcher (CD). All participants were given an information sheet about the project and signed a consent form prior to the focus group hui commencing.

### Kaimahi focus groups

The focus group hui were each conducted by a Māori researcher (JS). As they were conducted soon after the 2020 lockdown was lifted - when in-person communication was again possible - both focus groups were conducted kanohi-ki-te-kanohi at the Wainuiomata Community Hub. They lasted from 60 to 90 min. Once whānaungatanga (connection) was established, data were collected through a semi-structured interview questionnaire (available from the authors on request). Questions explored the kaimahi’s experiences of service provision leading up to, and during the nationwide lockdown. They were asked to reflect on their knowledge and experiences to inform how future health and social service responsiveness and resourcing could be enhanced. Each focus group was recorded and transcribed, and field notes were recorded. Separate ethical approval was obtained for the focus group hui from the University of Otago Human Ethics Committee (Ref No. D20/156).

### Data analysis

Data were coded and analysed thematically [27]. The kaimahi focus group transcripts were collaboratively coded and analysed by two Māori researchers (CD and CT-P) and the whānau transcripts were coded and analysed by three Māori researchers (CD, DL and CT-P) using NVivo 12 and Zoom video meeting technology. Individually, the authors familiarised themselves with the participant’s stories (data) by reading and re-reading the transcripts and listening to the audio-recordings and each began to develop codes. Following this familiarisation process, the initial codes were discussed together, ideas shared and the codes built upon through kōrero (discussion). Working in this way ensured thorough understanding and description of the data and reduced bias. As new transcripts were coded these were compared with previous transcripts to find similarities and differences then cross-coded to make links between the transcripts and deepen the coding frame. Codes were compared and contrasted then grouped by related concepts. Additionally, researcher field notes were analysed iteratively for themes and sub-themes. Through this process, repetitive themes were identified. These themes were discussed with the wider research team and final themes determined. The final themes aligned strongly to the long-standing Māori worldview concepts of kaitiakitanga, whānau and manaakitanga which are also integral to the values framework of Kōkiri Marae Hauora (further discussed in strengths and limitations).

## Results

The findings are presented below within the three overarching themes, kaitiakitanga, whānau and manaakitanga. Participants’ quotations are used to illustrate key points. Participant coding is as follows: W=Whānau, K=Kaimahi, RK = Rangatahi Kaimahi.

### Kaitiakitanga (guardianship)

Mā mua ka kite a muri, mā muri ka ora a mua.

*‘Those who lead give sight to those who follow, those who follow give life to those who lead’.*

Kaitiakitanga describes the concept of guardianship - often referring to guardianship over the environment - however, in this context it describes how Māori health care providers protected, unconditionally, their whānau (clients) in the community that they serve.

For all the participants, the feeling of being taken care of and supported by Kōkiri health and social services during the lockdown was evident. One participant spoke about feeling extremely lucky to have Kōkiri and the pātaka kai.

*“Oh … you know we are so lucky I say that about the whole of Wainui [omata]. If it wasn’t for Kōkiri I don’t know where the people here would turn to, because you know food banks that you couldn’t get into because you had to have a criteria to meet, but Kōkiri just opened their doors here and they just fed the people and sanitising packs, they just looked after the kaumātua and we are alright, we are alright!!”* (W:4)

Other participants commented about feeling proud to be Māori during the lockdown because of the work undertaken by Kōkiri to support people of all ethnicities.

“I couldn’t be more proud to be a Māori at this time, during COVID, because as we looked around and saw the Pākehā ones, it was like the Māori people had put their aroha [love] right up there and served the people. In the end, Kōkiri was there for the Māori people but they serviced everybody, there were no boundaries, no ethnicity, just if you needed a kai, it was right there. So, we were so lucky.” (W:2)

Some participants indicated that this was the first time they had accessed Kōkiri’s services and how much they appreciated the support they received.

“I have never been a part of this Marae ever or never supported it but ever since COVID I’ve always sworn that I’ll always tautoko [support] Kōkiri Marae. During COVID, they helped with so much whānau and all the mahi behind the scenes.” (W:1)

Kaimahi provided countless examples of their resourcefulness in navigating and supporting whānau with a wide range of needs. All kaimahi and rangatahi kaimahi spoke of “wrap around” support, “working outside of the square” and the vast range of support packages available for their community during the lockdown.

“We had tangi packs, kōhanga packs, kaumātua packs, motel packs, we had all these different kinds of packs for our community.” (K:3)

Kaimahi also commented that the approach they were able to take to meet the range of community needs was made possible because of Kōkiri’s strong leadership. They described the courage it took to establish - during a pandemic lockdown - the vast level of community support provided by Kōkiri, and the trust of the community and kaimahi in their leadership.

“One awesome thing about our leader, Teresea, she has that kind of insight. I couldn't see that at the start, so honestly, we would not have done that food bank if it wasn't for her faith. I remember saying, we just did 500 parcels in the last 3 days, how the hell can we keep this going? I thought oh my god, but you know, we listen to her, she has a lot of wisdom and now she has created this whole kaupapa, the Pātaka Kai.”(K:1)

### Whānau

Ehara taku toa i te toa takitahi, engari he toa takitini.

*‘My strength is not that of an individual but that of a multitude’.*

It is not by chance that whānau are at the centre of the Kōkiri values framework. The framework is a model with values that when woven together provided the foundation for kaimahi to work alongside whānau using a strengths-based approach, with whānau at the centre of all service delivery.

Kōkiri Marae was acknowledged by all the participants for the role they played ensuring their communities were in ‘safe hands’. The one Pākehā participant went so far as saying he now regarded Kōkiri workers as his whānau.

“I got the food box from [Kōkiri] but it didn’t end there, they would stop me on the street, they would keep the distance and talk to me on the street and ask me how I was going and they were just an extra family. What more can I say, I class them now as virtually my family. I can’t say anything else.” (W:6)

One woman living on her own was afraid to go outside of her property, she spoke about the community health worker and asthma nurse dropping off food parcels and then her asthma medication.

“Whaea Ann she got hold of me, I just didn’t want to go anywhere, so I got her to get hold of my GP. When she dropped off my food, I asked Aunty Huia - can you ask whaea Annie about some asthma pumps, she brought food first then asthma pumps.” (W:10)

Other participants spoke of the difficulties of working within the rules of lockdown, especially the ‘one size fits all’ approach to basic needs such as only allowing one person to shop per household. In this case, Kōkiri provided food parcels and shopped for other essential items so that this whānau member did not need to go out to the grocery store.

“Yes, I don't know how I would have managed. I heard stories of solo mums’ during lockdown, I don't know how I would have done that because I would have needed to take five of my children to the supermarket and I don't know how that would have worked.” (W:3)

However, lockdown also had a significant and positive impact on participants’ relationships. The majority of kaimahi and whānau interviewed responded positively about relationships that were strengthened because of being able to spend ‘unrushed time’ together.

“*You get rushed and you don’t actually get to have those kōrero with your children. It was good, it was positive. We could sit and enjoy each other instead of doing the day-to-day routine. We get home at 5.00 and then dinner, after dinner the dishes and after dishes, baths and in bed by 7.30. Everything is clockwork and that drains everyone.” (W:5)*

### Manaakitanga

He taonga rongonui, te aroha ki te tangata.

*‘Kindness towards people is a great treasure’.*

Manaakitanga encompasses reciprocal hospitality and mutual respect between individuals or groups of people. It describes that all living things should be treated with respect and that in turn they should treat others with respect, kindness and care regardless of the situation, and without judgement.

One participant provided a home for her son’s friend during lockdown. She spoke of how the asthma nurse had been looking for this young man and how relieved the nurse was to know he had found somewhere to live.

“So I rung Kōkiri up and spoke to this lady that was doing the asthma there and she knew him! She wondered where he was and I said, “well he’s living with us during COVID!” So she got him kai boxes because his benefit got stuffed up, so he was literally homeless for about three weeks before he came to us. Yeah, and Kōkiri helped get him bedding while he was staying with us and all his medications cos he needed pumps and stuff. She’d come over once a week to see him.” (W:8)

Whānau participants also spoke of the immense support that Kōkiri provided across the whole community.

“Kōkiri were amazing I don't know how to express that in words, the time and effort they put in. I watched it on social media, the Pātaka Kai and how they made sure that the community was taken care of and they had everything that they needed.” (W:7)

Many whānau said that Kōkiri was not only their main mechanism for support but also their primary mechanism for up-to-date and trusted information. Kōkiri’s social media platforms were especially noted, as was the approach taken by kaimahi when they were out in the community.

“Kōkiri was the main support for our whānau during COVID. The Kōkiri Facebook page is how I kept in contact with what was happening with COVID, they did daily updates, their page was just an awesome way to stay connected with the wider community, the videos that got posted up, the information they posted, the food parcels and the sanitation packs that appeared on your doorstep, and always with a kia ora, a wave and a smile.” (W:3)

A kaimahi spoke of confusion within their communities about whether people could see their General Practitioner. When delivering kai parcels, kaimahi would always ask whānau about their health and discuss any other support they may need.

“We dropped a parcel to a whānau and she had swollen feet that had gone black, really badly swollen feet and so we got out our camera. I said, ‘I'm sorry whaea, I need to take a photo of your feet’. We were fortunate that Kōkiri had Doctor Alice, a doctor that you could refer our whānau to. We weren't just a food bank we had doctors and nurses and social workers.” (K:2)

Kaimahi reinforced manaakitanga as being a fundamental value that guided their practice - both individually and as a team.

“I believe we are a very tight team and even through Covid it's just got more solid, the solidarity, the aroha is there. It's been an amazing experience although a sad kaupapa but it's been a really neat experience for us as a rōpū of kaimahi, I would do it again in a heartbeat, awhi mai, awhi atu” [when we show care to others, they will return that care to us].” (RK:1)

Manaakitanga was also reflected in the feedback from the whānau who spoke about giving back and paying forward. Essentially the whānau replicated the manaakitanga that was shown to them by Kōkiri and its kaimahi and paid it forward to others in their community.

“Kōkiri Marae gave to us and so I gave that neighbourly support. Yelling out you know “are you ok”. I went around to all the widows and made sure that they were ok and if not I made sure that they got food boxes and hand sanitising boxes and stuff. It was more the elderly that I was concerned about. It wasn't just about us, it was about our community.” (W:10)

## Discussion

This study explored an urban marae-led response to the nationwide 2020 COVID-19 lockdown and the impact of their contributions to address the health and social support needs of their whānau. It did so through qualitative research with whānau and kaimahi within the Wainuiomata and Hutt Valley regions of Aotearoa New Zealand. The study identified that the long-standing Māori world values of kaitiakitanga (protecting) and manaakitanga (with kindness) - with whānau at the centre of all decisions and service delivery - worked as a protective mechanism to provide much-needed support within the Wainuiomata community during Aotearoa’s 2020 lockdown.

It is evident from this study that Kōkiri provided a significant amount of support during the 2020 lockdown across the Greater Wellington (including Wainuiomata) communities. In keeping with a Māori worldview and the concept of kaitiakitanga this support was unconditional and universal, including for non-Māori whānau. Many whānau participants described enormous appreciation for Kōkiri and the many instances of kaitiakitanga provided by kaimahi. Importantly, the kaitiakitanga approach appeared to infuse a sense of reassurance and in turn reinforced the participants’ ability to build resilience and to care for their own whānau. Building resilience in a community especially when whānau are isolated and at risk of illness or further financial stress is crucial [28] and emphasises the key role that Māori services play in working alongside vulnerable communities to support their wellbeing in culturally meaningful ways [28–30].

Manaakitanga - which also encompassed many facets of Kōkiri’s service delivery - shows how Māori communities care for and support one another and demonstrates the tribal, community and whānau supports for both individuals and whānau [31]. Others also identify the significance of relationships and the crucial role that relationships play in engendering trust thus enabling whānau to draw on the resources of the community, especially in times when Māori are vulnerable [15, 16, 29]. For many participants, the long-standing relationships between Kōkiri and whānau were key to feeling and being supported. For others, the embodiment of manaakitanga - through no criteria to meet, no forms to fill in, no limits placed on support provided - and the uniquely Māori service delivery model meant that trust was able to be quickly established. Strong leadership underpinned by Māori values - identified as a critical success factor in other studies [15, 16, 29, 32] - was also evident.

However, kaitiakitanga and manaakitanga while an integral part of Kōkiri’s values framework only work when whānau are at the centre of all service delivery. Māori health providers know that the whānau-centred approach based on Māori values works well within the communities of which they are a part, including for non-Māori whānau [15, 32, 33]. As with Iwi or Māori health providers during other public health crises, Kōkiri was well placed to determine and provide for the wellbeing of whānau in their community [15, 16]. Māori providers are deeply cognisant of the health and social complexities of whānau within their communities [34] and the significant inequities in healthcare access [32, 33]. Like other providers, Kōkiri quickly recognized that crisis response services were not meeting the needs of their community and were easily able to develop, adjust and quickly mobilise their services to meet that need [15, 16]. Kōkiri had the infrastructure needed to make initial phone calls to their client lists to assess community need. They utilised their own Māori-specific communication strategy through their Facebook page and website and used their established relationships with Government and non-Government funders to bring support in. Then together with other relevant organisations Kōkiri implemented a local Māori pandemic response that met the needs of whānau in their community.

Importantly, a wide range of health-related services was provided by Kōkiri and often the pātaka kai was the gateway to these services, allowing Kōkiri kaimahi to assess and meet the varied needs of whānau. While the bulk of the support was discontinued once the 2020 lockdown ended, the pātaka kai continues as a foodbank and community resource within the Wainuiomata community [35]. The Māori concept of utu (reciprocation or balance) is a demonstration of giving back and is intrinsically linked to manaakitanga; to retain mana (power or prestige) all actions (both friendly and unfriendly) require an appropriate response. Establishing the pātaka kai gave the rangatahi kaimahi the opportunity to both support Kōkiri and to give back to their community. Now the pātaka kai gives back to the community in which the kaimahi live through add-on initiatives, including developing a community garden, providing education and training in gardening and cooking, providing a safe drop-in meeting place, and more. Giving back (and paying forward) is for many Māori a cultural obligation [17], whereby an individual cannot hold on to something, rather it must go to where it is needed. For the Wainuiomata community, the pātaka kai became a space for utu to happen. Cram [36] similarly illustrates the extra activities that Māori health providers practice in challenging times. While this is described as ‘mahi aroha’ or work done out of a love for the people, Cram further describes how mahi aroha works to uphold the mana of others and also to enhance the mana of kaimahi through their acts of care [36].

Kōkiri has worked alongside their community over the past three decades [2]. The establishment of the pātaka kai was feasible because of Kōkiris’ strong leadership, the existing relationships they have built up over time and their in-depth knowledge of and credibility within their community. However, it was also made possible as the Government funders - such as Ministry of Health, the Whānau Ora Commissioning Agency and District Health Boards, who are the primary source of funding for many Māori providers [33, 37] - removed barriers and broke down previously siloed funding, allowing providers to more quickly respond to the many and urgent needs of vulnerable communities [30].

The Māori-centred and initiated response to a pandemic described in this paper demonstrates the effectiveness of building on existing Māori community strengths and of sharing power and resources with Māori health providers [38]. However, Māori providers remain undervalued and under-resourced within the health system today [39–41] as they are impacted by the structural bias and institutionalised racism that pervades within New Zealand society [17, 41–43]. Despite this, Māori health providers play a substantial role in supporting their communities underpinned by strengths-based and mana-enhancing values while also building resilience within these communities [30, 38]. In this way, Māori knowledge and enhanced capability of iwi and Māori health providers will help to ensure that society is more responsive to vulnerable whānau and the system is more effective at protecting community wellbeing in a public health crisis.

## Limitations

There are several limitations of this study. First, the participants were known to the researchers (especially the lead researcher CD who is a Service Manager at Kōkiri). While this degree of familiarity could be seen as a potential cause for bias, it is very common for Māori within a region the size of those where the research took place to be familiar with or related to each other. We attempted to reduce any bias by utilizing an Otago University employee (JS) to conduct the kaimahi focus group hui, rather than a direct work colleague (CD). However, this level of familiarity can also be thought of as a strength of the study. It is possible that whānau members were more likely to share information and details with the researchers, due to the level of trust in Kōkiri. Additionally, this type of participatory research - that needs high trust - would not be able to be conducted except by Kōkiri themselves who are part of the community and understand the lived reality of many whānau. Second, while we started out using an inductive approach to data analysis, we determined through the analysis phase that the themes being formed were within the values framework of Kōkiri Marae Hauora. The long-standing Māori worldview concepts of kaitiakitanga, whānau, and manaakitanga are integral to the values framework, Kōkiri kaimahi are trained in the framework and operate under its values as their practice model, and so it is understandable that these values were reflected by kaimahi and whānau and also seen by those analysing the data. Third, while this study has a relatively small sample associated with one Māori health provider, it was not meant to be representative or generalisable. Rather, it was designed to provide valuable insight and in-depth information into the role of that provider, how their values guided their Covid-19 response and thus how they were able to support their whānau and mitigate the impacts of Covid-19 s’ response on a vulnerable community. Finally, while we conducted the research including interpretation of the findings from an indigenous Māori worldview, there is diversity in views and values of those who identify as Māori. However, the values discussed in this paper are long-standing Māori values so we believe that the findings will be applicable to other Māori organisations and to other indigenous populations who often share similar values. Additionally, we believe the saying that ‘what is good for Māori, is good for all’ and so delivering critical community services with kindness and approaches that uphold mana will also be good for non-Māori communities.

### Recommendations

This study provided a valuable forum for researchers, kaimahi, and whānau to come together to reflect on their experiences of the 2020 lockdown and the Kōkiri Marae response. Based on these insights, we propose the following recommendations for the future:

- Māori-led pandemic responses. It is essential for iwi, Māori and Māori health providers to be included in pandemic planning and decision-making [44]. The current Covid-19 vaccination rates - with Māori showing the lowest rates by ethnicity [45] - are a case in point. Inclusion of Māori approaches and priority for Māori early in Aotearoa’s vaccination campaign would have averted the lower vaccination rates and higher case likelihood observed for Māori in the August 2021 Delta outbreak (35% of cases) [46–48].
- Autonomy and appropriate resourcing. Māori-led initiatives and organisations must be valued and adequately resourced to enable critical ‘by-Māori for-Māori’ support at all times, but especially within a public health crisis. As identified in other studies [29], providers are able to quickly and effectively respond to community need when devolution of funding along with release from contractual obligations gives providers the autonomy to determine how resource is distributed.
- Strong communities with strong networks. During non-crisis times, communities and providers should be connected to the resources they need so that they can create and further strengthen support networks [29]. Ensuring that these networks are in place and ready to sustain community wellbeing in an emergency (such as the ongoing legacy of the pātaka kai) before disaster strikes will help to mitigate negative impacts of any future emergency.
- Kaupapa Māori research. More Māori-led research such as this study is crucial to better understand how to meet the health and wellbeing needs of whānau in uniquely Māori ways.

## Conclusion

It was easy for Kōkiri kaimahi to take on the message of manaakitanga advocated by Aotearoa’s Prime Minister, as manaakitanga was already enshrined in the values framework within which they practice. With whānau at the centre, kaitiakitanga (protecting) and manaakitanga (kindness) have worked together to protect whānau and soften the worst impacts of Aotearoa’s 2020 lockdown among the Wainuiomata community. A public health crisis - such as that presented by COVID-19 - is when whānau need protecting the most. It is also when a Māori provider as a trusted member of the community is invaluable. We believe that Māori health providers are a tāonga (treasure) and are very well placed to respond effectively in a crisis when resourced appropriately and trusted to deliver.

## Supplementary Information

**Additional file 1.**

**Additional file 2.**

## Data Availability

The transcripts used and/or analysed during the current study are not available for external purposes.
