# Supplementary material for: A kia ora, a wave and a smile: an urban marae-led response to COVID-19, a case study in manaakitanga
Source: Int J Equity Health. 2022 May 17;21:70. doi: 10.1186/s12939-022-01667-8 (PMC9112650; doi:10.1186/s12939-022-01667-8)
Supplement: Supplementary file 1 — Additional file 1. [file 12939_2022_1667_MOESM1_ESM.docx]

Additional file 1

**Glossary**

aroha - love

kai - food

kaimahi - workers

kaimahi - worker

kanohi-ki-te-kanohi - face to face

kaupapa Māori - literally 'a Māori way', connected to Maori philosophy and principles

kia atawhai - Be kind

karakia - prayer

kia ora - hello

kōrero - discussion

koha kai - gift of food

koha - a gift, offering, donation, contribution used to preserve connections and has implications of reciprocity.

mahi aroha - work done out of a love for the people

manaakitanga – caring

mana- prestige

mana-enhancing - a way of engaging with others that enables them to retain their power and prestige

manaakitanga - kindness, support, or the process of showing respect and care for others

marae - a traditional meeting place that is still a vital part of everyday life for contemporary Māori

pākehā – New Zealand European/ a Māori-language term for New Zealanders primarily of European descent

pātaka kai - foodbank and community food resource

rangatahi - youth

tangi – funeral

tāonga - special treasure or a gift

tautoko - support

te ao Māori - the Māori world view

Te Rūnanga o Ngāi Tahu - a Māori tribal authority in the South Island of Aotearoa New Zealander

tikanga –Māori customary practices, behaviours or protocol

whānaungatanga – relationships, connection

whaea - respected elder woman

whakatauki - a saying with deep philosophical meaning
