# Supplementary material for: A kia ora, a wave and a smile: an urban marae-led response to COVID-19, a case study in manaakitanga
Source: Int J Equity Health. 2022 May 17;21:70. doi: 10.1186/s12939-022-01667-8 (PMC9112650; doi:10.1186/s12939-022-01667-8)
Supplement: Supplementary file 2 — Additional file 2. [file 12939_2022_1667_MOESM2_ESM.docx]

Additional file 2

**Figure 2: Kōkiri Marae Hauora Values Framework**


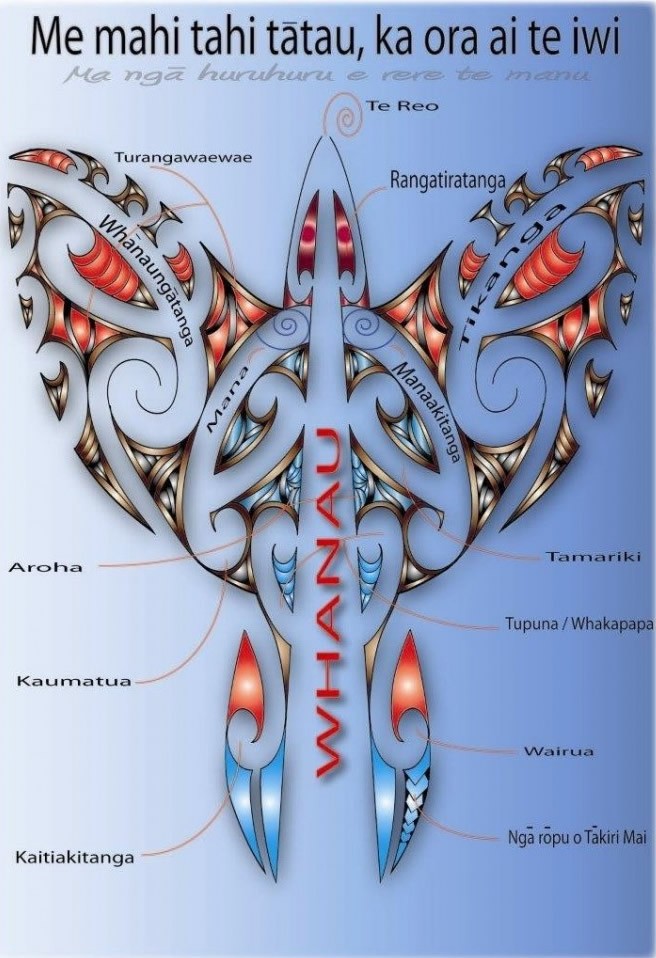


Kōkiri can be perceived as a manu (bird) and the huruhuru (feathers) belong to the Kōkiri workforce, whānau and the community. It is the way the feathers are aligned and work together that allows the manu to fly, and thus the whānau to thrive.

The core values within the framework are the underpinning principles and beliefs of Kōkiri that when woven together provide the foundation for kaimahi to work alongside whānau. The core values ensure services are delivered in a consistent mana-enhancing way, build on whānau strengths and capability and support the development of whānau leadership, and thus enable the best outcomes for whānau to be achieved. The values are described in Table 1 below.

**Table 1: Description of Kōkiri values**

| Value | Description |
| --- | --- |
| Aroha (Love) | All whānau members are born with the right to be cared for and loved unconditionally |
| Kaitiakitanga (Guardianship) | All living things enhance, protect and sustain their own sense of wellbeing and the wider environment they live in |
| Kaumātua (Elder) | Kaumātua are storehouses of knowledge, wisdom and experience |
| Mana (Power, Respect) | All living things have mana, an intrinsic quality that manifests in them achieving their dreams and aspirations |
| Manaakitanga (Caring) | All living things are treated and treat other with respect, care and regards, regardless of the situation |
| Rangatiratanga (Autonomy) | All whānau members determine their own future individually as well as a whānau collective |
| Tamariki (Children) | All children are born with a purpose in life |
| Tikanga (Protocol) | All whānau are supported to maintain and uphold their own practices and protocols that are unique to their own whānau |
| Tūpuna (Ancestors) | All whānau members are supported by their tupuna |
| Turangawawewae (place where one has the right to stand) | All whānau members have a place to belong and are respected as a representative of their whānau whakapapa |
| Wairua (Spirit or soul) | All whānau members are born with the capacity to sustain and embrace their own wairua |
| Whānaungatanga (relationships) | All whānau members are connected and actively contribute to building healthy relationships within their whānau, community and society |
